# Crosstalk Between Opioids and the Anti-Tumour Immune Checkpoint Axis

**DOI:** 10.3390/curroncol33070411

**Published:** 2026-07-09

**Authors:** Parsa Alan, Marie-Odile Parat

**Affiliations:** 1 Faculty of Medicine, The University of Queensland, Brisbane, QLD 4029, Australia; 2 School of Pharmacy and Pharmaceutical Sciences, The University of Queensland, Brisbane, QLD 4102, Australia

**Keywords:** immune checkpoint inhibitors, opioid, PD-1, PD-L1

## Abstract

Opioids are frequently prescribed for cancer pain management, yet accumulating evidence suggests that opioid exposure may be associated with inferior outcomes in patients also undergoing treatment with immune checkpoint inhibitors (ICIs). To synthesize mechanistic and clinical evidence linking opioids to the PD-1/PD-L1 axis, the literature was searched up to 18 January 2026, with study selection and data extraction focused on (i) cancer-cell and immune-cell effects of opioid agonism or antagonism on PD-1/PD-L1 biology, and (ii) clinical studies reporting ICI outcomes (progression-free survival, overall survival, or treatment duration) with concomitant opioid exposure. Preclinical studies support multiple, non-mutually exclusive mechanisms: opioids can induce PD-L1 in tumour cells, modulate innate-inflammatory pathways (including TLR4-linked cascades), promote dysfunctional T-cell phenotypes that reduce responsiveness to PD-1 blockade, and show context- and opioid-dependent effects. Clinical cohorts and meta-analytic datasets in non-small cell lung cancer and other tumour types report associations between opioid exposure (including higher morphine-equivalent dosing) and worse ICI outcomes. The intersection of opioid signaling with PD-1/PD-L1 biology likely operates across cancer cell-intrinsic and immune cell-intrinsic pathways, providing a mechanistic rationale for prospective evaluation of opioid-sparing strategies and/or peripheral opioid antagonism as adjuncts to checkpoint blockade.

## Simple Summary

Tumours manipulate the immune system to their benefit. Immune checkpoint inhibitors are anti-cancer drugs that restore the ability of the immune system to eliminate tumours. These drugs have shown efficacy through increased survival for many cancer patients. Opioids are analgesics commonly given to cancer patients to alleviate disease-related pain. A growing number of studies suggest that patients who take opioids while receiving immune checkpoint inhibitors may not respond to treatment as well as those who do not. Opioids may change cancer cells and immune cells in ways that weaken the immune response these therapies rely on. In this review, we bring together laboratory and clinical findings to explain how opioids and this immune pathway might interact. Our aim is to highlight questions for future research, including whether reducing opioid use or blocking certain opioid effects could improve responses to immunotherapy.

## 1. Background

Inhibitors targeting the Programmed Cell Death Protein 1 (PD-1), Programmed Cell Death Ligand 1 (PD-L1) or Cytotoxic T-lymphocyte associated protein 4 (CTLA-4) immune checkpoints have fundamentally altered the treatment landscape for multiple malignancies. ICIs function to produce durable responses across several tumour types by blocking immunoinhibitory signals and restoring an effective anti-tumour response [1,2]. Targeting the immune checkpoint protein lymphocyte activation gene 3 (LAG-3) has recently entered clinical practice as an adjunct to PD-1 blockade [3]. Despite these advances, primary resistance and treatment failure remain common, even in tumours with favorable biomarkers such as high tumour mutational burden or baseline immune infiltration [4,5]. This variability in response has prompted increasing interest in host-related factors that may influence immunotherapy efficacy independently of tumour genomics.

ICIs are monoclonal antibodies used in cancer immunotherapy to target immune checkpoint proteins found on the surface of tumour and immune cells. In cancer progression, cancer cells evade host detection through immune checkpoint protein-mediated inhibitory signaling. ICIs have been developed to restore and enhance anti-tumour immune function through selective modulation of this signaling tone, achieving an effective anti-tumour T-cell response [1]. To date, there are several FDA-approved ICIs including CTLA-4 monoclonal antibodies ipilimumab and tremelimumab, PD-1 monoclonal antibodies nivolumab, pembrolizumab, cemiplimab, dostarlimab, retifanlimab, toripalimab, and tislelizumab, and PD-L1 monoclonal antibodies atezolizumab, durvalumab, and avelumab [3]. An additional anti-PD-1 antibody sasanlimab is currently in phase 3 clinical development [6].

Inhibiting CTLA-4 and PD-1 has similar results on T-cell activity, with the nuance that targeting CTLA-4 helps with priming new T cells in the lymphoid organs, while PD-1, expressed on activated T cells as well as B cells and myeloid cells, signals predominantly within the tumour microenvironment, during the effector phase [7]. PD-L1 on tumour or immune cells engage with PD-1 receptors on T cells to suppress T-cell-mediated cytokine production and cytotoxicity. ICIs targeting either PD-1 or PD-L1 therefore prevent cell-mediated inhibition of immune function within the tumour microenvironment [8]. In the clinic, ICIs are administered intravenously to target multiple tumour types using either fixed or weight-based dosing based on patient characteristics. ICI therapy is often combined with chemotherapy, radiation therapy, other anti-cancer therapies, or analgesic medicines [1,9]. Although ICIs can produce durable tumour control and long-term survival in a subset of patients, their efficacy depends on intact host immune function, rendering immunotherapy outcomes potentially sensitive to concurrent host- and treatment-related modulators of immunity.

Many cancer patients experience pain at different stages of their disease; for example, in the perioperative period, as a result of therapy, as the cancer grows, and in palliative care. Opioids are key to the management of moderate to severe pain associated with cancer [10]. Their analgesic activity is primarily via μ-opioid receptors (MOR) within the central nervous system after passing the blood–brain barrier (e.g., morphine, fentanyl, oxycodone, and hydromorphone) where they block nociceptive pathways. Opioid receptors are also expressed in peripheral tissues, for example on immune, endothelial as well as cancer cells, where they can be engaged by systemic opioids. Peripherally acting antagonists such as methylnaltrexone, naldemedine, or naloxegol can antagonize MORs in peripheral tissues while sparing central analgesic pathways due to their impermissible transfer through the blood–brain barrier [11]. The relative contribution of central versus peripheral mechanisms, as well as immune-modulatory effects, varies by opioid type, receptor selectivity, pharmacokinetics, dose, duration of exposure, and clinical context which collectively underscore the heterogeneity of opioid–immune interactions in cancer patients.

Against this backdrop, several retrospective clinical studies have reported associations between opioid exposure and worse survival outcomes in patients treated with ICIs across multiple cancer types and particularly in non-small cell lung cancer (NSCLC), raising the possibility that opioid signaling may intersect with pathways critical for effective anti-tumour immunity under checkpoint blockade [12,13,14,15,16,17,18,19,20,21,22,23,24,25,26,27]. However, these associations remain largely observational, and disentangling direct immunological effects of opioids remains an important challenge.

In many of these clinical studies, opioid exposure is treated as a binary, non-adjustable variable, with limited reporting of dose, duration, timing relative to ICI initiation, or opioid class, amongst other factors. Further, immunotherapy regimens are frequently heterogeneous, often administered in combination with chemotherapy or other agents, with incomplete reporting of dosing and treatment duration, thereby making conclusive statements immature.

For the reasons outlined above, the impact of ubiquitous concomitant medications such as opioids on immunotherapy outcomes warrants scrutiny. Given the widespread and sometimes unavoidable use of opioids in oncological pain management, it remains unclear whether these associations simply reflect advanced disease and symptom burden or instead indicate active modulation of anti-tumour immunity. The convergence of emerging clinical signals with mechanistic, transcriptomic, and preclinical evidence therefore invites a timely integrative review. Accordingly, this review synthesizes experimental, transcriptomic, and clinical data to develop a coherent framework connecting opioid pharmacology to immune checkpoint regulation within the cancer context.

## 2. Methods

The following literature search strategy was applied up to 18 January 2026 using Pubmed:

(opioid* OR “Opioid”[MeSH Terms] OR morphine OR fentanyl OR buprenorphine OR tramadol OR oxycodone OR hydromorphone OR methadone OR codeine OR heroin OR alfentanil OR remifentanil OR pethidine OR meperidine OR tapentadol OR nalbuphine) AND (PD1 OR PD-1 OR “Programmed Cell Death 1 Receptor”[MeSH Terms] OR “programmed death 1” OR “programmed death-1” OR CD279 OR nivolumab OR pembrolizumab OR Keytruda OR BGB-A317 OR PDL1 OR PD-L1 OR “programmed death ligand 1” OR CD274 OR atezolizumab OR avelumab OR durvalumab OR CTLA4 OR CTLA-4 OR “cytotoxic T-lymphocyte-associated protein 4” OR ipilimumab OR Yervoy OR tremelimumab OR Imjudo OR abatacept OR Orencia OR belatacept OR Nulojix OR checkpoint*).

**Figure 1:**
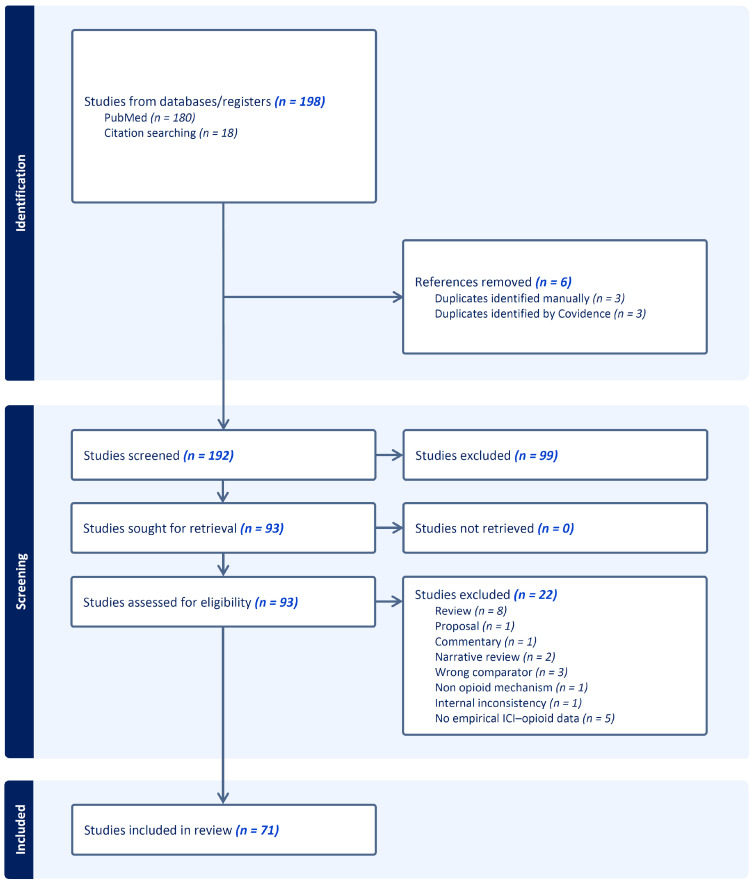
PRISMA flow diagram summarising study identification, screening, eligibility assessment, and inclusion for the review.

Records retrieved were imported into Covidence. Study selection was conducted in accordance with the predefined eligibility criteria. Title and abstract screening, followed by full text review, were performed using the exclusion workflow outlined in Figure 1. Reasons for exclusion at the full text stage were documented to ensure transparency and reproducibility of the selection process. Additional studies were included where needed for completeness of this review.

## 3. Clinical, In Vivo, and In Vitro Studies Linking Opioid Exposure to Immune Checkpoint Blockade

### 3.1. Clinical Evidence

Across tumour types, accumulating clinical evidence suggests that opioid exposure is associated with worse outcomes in patients treated with ICIs, including reduced progression-free survival (PFS), overall survival (OS), or treatment durability (Table 1). Multiple retrospective cohorts and one prospective cohort in advanced non-small cell lung cancer (NSCLC) report significantly worse PFS and/or OS among patients receiving opioids during or around ICI therapy [12,14,16,18,19,22,23,27,28]. Similar associations have been observed in mixed solid tumour and non-NSCLC patient populations, where concomitant or strong opioid use has been linked to shorter survival outcomes during PD-1/PD-L1-based immunotherapy [13,15,17,20,24]. A recent meta-analysis focused on NSCLC further supports this pattern, demonstrating pooled hazard ratios of approximately 2 for both PFS and OS among opioid-exposed patients receiving ICIs [21], and results from another meta-analysis focused on urothelial carcinoma also followed this trend [29].

The association between opioid exposure and worse outcomes with ICI therapy is supported by retrospective analyses and meta-analytic signals in several tumour types (Table 1). Importantly, in addition to binary opioid exposure (yes/no) used in many studies, some cohorts reported a dose–response pattern in which greater opioid utilization (including higher morphine-equivalent dosing strata) was associated with markedly shorter immunotherapy treatment duration and overall survival, reinforcing the clinical relevance of opioid–ICI antagonism even as residual confounding by disease severity and pain burden remains possible [20,22]. Of note, one clinical study found that high opioid use was positively associated with increased PD-L1 expression in NSCLC patients receiving ICI therapy [30].

Notably, in a retrospective study of advanced biliary tract cancer receiving cisplatin, gemcitabine and durvalumab cancer therapy, patients receiving analgesics that include a combination of non-steroidal anti-inflammatory drugs (NSAIDs) and/or opioids exhibited no significant change in survival outcomes with analgesic use [26]. One retrospective study with stage IV NSCLC patients receiving monotherapy showed no significant association of survival outcomes with opioid exposure as an independent variable [25].

As previous reviews suggest, these clinical associations do not, in themselves, establish a direct causal effect of opioids on immune checkpoint pathways. Most available data derive from retrospective observational cohorts, where opioid use may act as a surrogate for more advanced disease, higher symptom burden, poorer performance status, or palliative intent, all of which independently predict worse outcomes. While several studies employed multivariable adjustment or propensity-based methods to mitigate confounding, residual confounding by indication remains likely [13,26,28]. Furthermore, many clinical cohort studies assess opioid exposure as a medication variable rather than directly measuring PD-1, PD-L1, or CTLA-4 expression or signaling in patient immune or tumour compartments. Prospective studies incorporating longitudinal immune profiling, both ICI and opioid dose and duration metrics, and mechanistic correlates are therefore warranted to clarify whether opioids directly modulate immune checkpoint function and whether opioid use primarily reflects underlying disease severity and clinical trajectory.

**Table 1:** Clinical studies examining opioid exposure and outcomes in patients treated with immune checkpoint inhibitors.

		Univariate/Unadjusted			Multivariate/Adjusted			Univariate/Unadjusted			Multivariate/Adjusted					
Study	Cancer Type	PFS or Equivalent	95% CI	*p*-Value	PFS or Equivalent	95% CI	*p*-Value	OS	95% CI	*p*-Value	OS	95% CI	*p*-Value	Opioid and Length of Treatment	ICI Class (Dose if Available)	Study Population and Source
Bironzo et al. (2019) **[12]**	Advanced NSCLC	4.16	2.15–8.05	<0.001	3.19	1.45–7.01	0.004	4.68	2.09–10.52	<0.001	4.16	1.61–10.76	0.003	Patients receiving opioid at ICI start (mean MEDD of 59 mg)	Monotherapy of anti-PD-1 or anti-PD-L1 (dose NR)	Multicenter retrospective study, *n* = 75 total (Italy)
Cortellini et al. (2020) **[13]**	Stage IV NSCLC, melanoma, RCC	2.05	1.56–2.71	<0.0001	1.71	1.28–2.28	0.0002	2.14	1.58–2.91	<0.0001	1.53	1.11–2.11	0.0098	Opioid use reported as a concomitant medication at start of immunotherapy (timing/length NR)	Monotherapy of nivolumab (3 mg/kg IV biweekly or flat dose 240 mg q2w), pembrolizumab (200 mg IV every 3 weeks), atezolizumab (1200 mg IV every 3 weeks)	Multicenter retrospective cohort from several medical oncology departments, *n* = 1012 total (Italy), 2014–2020
Verschueren et al. (2021) **[14]**	Stage IV NSCLC	NR	NR	NR	NR	NR	NR	NR	NR	NR	2.27	1.24–4.16	<0.01	Opioid use reported if opioids used within a timeframe of 30 days before or after the start of either immunotherapy or chemotherapy	First, second-, or third-line immunotherapy (nivolumab, pembrolizumab, atezolizumab, dose NR)	Multicenter retrospective matched cohort study, *n* = 442 total (Netherlands), 2015–2019
Miura et al. (2021) **[16]**	Advanced NSCLC	NR	NR	NR	1.39 (TTF)	1.05–1.85	0.021	NR	NR	NR	1.54	1.12–2.11	0.007	Concomitant opioid use at start of immunotherapy	nivolumab (3 mg/kg each cycle) or pembrolizumab (200 mg/bodyweight each cycle)	Retrospective cohort of patients treated with ICI, *n* = 304 total (Japan), 2016–2018
Mock et al. (2021) ^†^ **[30]**	Stage IV NSCLC	NR	NR	NR	7.4 vs. 1.8 median months with high opioid use (DOT)	7.4 months (6.2–8.8), 1.8 months (1.1–2.3)	0.001	NR	NR	NR	14.5 vs. 3.8 median months worse OS with high opioid use	14 months (11.7–16.3), 3.8 months (3.0–4.8)	0.001	Any opioid prescription at start of ICI therapy or two weeks prior to treatment; MEDD > 50 considered high and MEDD < 50 considered low opioid use	NR	Retrospective cohort of NSCLC patients treated with checkpoint inhibitors *n* = 208 (USA), 2015–2020
Mao et al. (2022) ^¶^ **[31]**	NSCLC, melanoma	1.61 (1.37–1.89), *p* < 0.001						1.67 (1.30–2.14), *p* < 0.001						As defined within each study	Any ICI treatment	Meta-analysis of 11 retrospective studies *n* = 4404 total (Spain, Italy, Japan, France, USA), end date 2021
Yu et al. (2022) **[18]**	Advanced lung cancer	4.141	2.715–6.317	<0.001	4.994	3.217–7.753	<0.001	3.582	2.103–6.102	<0.001	3.618	2.030–6.240	<0.001	Any opioid exposure within one month of ICI therapy commencement	ICI monotherapy or ICI combination therapy (dose NR)	Retrospective cohort study with *n* = 132 total (China), 2019–2021
Taniguchi et al. (2023) **[19]**	NSCLC	1.17 vs. 2.07 median months (opioid vs. no opioid)	1.17 (0.93–1.73), 2.07 (1.23–4.73)	0.002	NR	NR	NR	4.2 vs. 9.57 median months (opioid vs. no opioid)	4.2 (2.53–6.20), 9.57 (2.23-)	0.018	NR	NR	NR	Median oral MEDD of 30 mg (7–180 mg range) with median treatment duration of 69 days prior to commencing nivolumab therapy	nivolumab (dose NR)	Retrospective, single-center cohort *n* = 296 total (Japan), 2015–2018
Guo et al. (2024) ^¶^ **[21]**	NSCLC	NR	NR	NR	2.16 for worse PFS with opioid use	1.26–3.71	NR	NR	NR	NR	2.02 for worse OS with opioid use	1.54–2.63	NR	As defined within each study	Any ICI treatment	Meta-analysis of 8 retrospective studies plus retrospective validation cohort *n* = 1174 total (Italy, France, Japan, Netherlands, China), end date 2023
	NSCLC	2.126	1.348–3.355	0.001	2.070	1.280–3.348	0.003	2.497	1.488–4.190	<0.001	2.808	1.635–4.821	<0.001	Any opioid (morphine, fentanyl, oxycodone, tramadol) before and/or during treatment	ICI alone or combined with chemoradiotherapy or targeted treatment (dose NR)	Retrospective validation cohort, *n* = 107 ICI-treated (of 181 total), China, 2018–2023
Hong et al. (2024) **[32]**	NSCLC, urothelial carcinoma, malignant melanoma	NR	NR	NR	NR	NR	NR	NR	NR	NR	NSCLC: 1.59, UC: 1.68, MM 1.57, worse OS with opioid use	1.49–1.70, 1.39–2.03, 1.23–1.99	<0.0001, <0.0001, <0.0001	Any opioid prescription within 30 days prior to initiating ICI therapy	First-, second- or third-line use of pembrolizumab, nivolumab, atezolizumab	Retrospective study of adult patients newly treated with ICI *n* = 8870 total (South Korea), 2017–2020
Young et al. (2024) **[22]**	Advanced NSCLC	3.87 (DOT)	2.52–5.95	0.0001	2.98 (DOT)	1.80–4.94	0.0001	3.84	2.51–5.87	0.0001	3.43	2.08–5.66	0.0001	High opioid use defined as MEDD ≥50 vs. low/no opioid use (MEDD <50); derived from opioid prescriptions 2 weeks before ICI initiation to ICI discontinuation	ICI monotherapy or ICI with chemotherapy (dose NR)	Retrospective, single-center cohort *n* = 209 total (USA), 2015–2020
Balcik et al. (2025) ^‡^ **[23]**	Metastatic NSCLC	4 vs. 8 median months (opioid vs. non-opioid)	4 months (1.72–6.28), 8 months (3.38–12.62)	0.006	3.166	0.413–24.254	0.267	7 vs. 14 median months (opioid vs. non-opioid)	7 months (3.56–10.44), 14 months (9.05–18.95)	0.03	0.798	0.096–6.618	0.835	Any documented use of opioids during nivolumab therapy (subcategorized by tramadol, morphine, fentanyl, codeine)	nivolumab (dose NR)	Multicenter retrospective across five hospitals, *n* = 209 total (Turkiye), 2018–2024
Brinzevich et al. (2025) **[28]**	Stage IV NSCLC	NR	NR	NR	1.23 TTNT with opioid use	1.14–1.32	<0.001	NR	NR	NR	1.28 worse OS with opioid use	1.18–1.38	<0.001	User vs. non-user classification based on ≥1 outpatient or inpatient opioid prescription within 3 months before ICI therapy	ICI monotherapy (pembrolizumab, nivolumab) or combination therapy (pembrolizumab with carboplatin or paclitaxel)	Multicenter retrospective study, *n* = 3739 patients receiving immunotherapy (USA), 2005–2023
Gobbini et al. (2025) **[33]**	Advanced NSCLC	1.41	1.21–1.63	<0.0001	1.28 for worse PFS with morphine use	1.09–1.50	0.003	1.47	1.26–1.71	<0.0001	1.35 for worse OS with morphine use	1.13–1.60	0.0007	Morphine (prescription from 90 days before to 30 days after first nivolumab administration)	nivolumab (3 mg/kg every 2 weeks)	Retrospective cohort with patients with advanced NSCLC who received in second or later lines of treatment, *n* = 753 (France), 2015
Liguori et al. (2025) ^†^ **[25]**	Stage IV NSCLC	decreased PFS with opioid use, ICI monotherapy	NR	0.0460 (log-ranked)	0.56 in opioid users vs. non-users with ICI monotherapy	0.26–1.20	0.1380	decreased OS with opioid use, ICI monotherapy	NR	0.0380 (log-ranked)	0.53 in opioid users vs. non-users with ICI monotherapy	0.22–1.30	0.1660	Any concomitant opioid use at start of ICI therapy	atezolizumab or nivolumab or pembrolizumab monotherapy or ICI combination therapy	Retrospective *n* = 200 approximate total adult lung cancer patients (Italy), 2016–2023
Cavaliere et al. (2026) **[27]**	Stage IV NSCLC	NR	NR	NR	1.99 in opioid users vs. non-users	1.03–3.83	0.0394	NR	NR	NR	2.66 in opioid users vs. non-users	1.35–5.24	0.0046	Any concomitant opioid use at start of ICI therapy	Anti-PD-1/PD-L1 monotherapy: pembrolizumab, nivolumab, cemiplimab, atezolizumab (dose NR)	Prospective *n* = 71 (total) stage IV NSCLC patients (Italy), 2019–2023

Iglesias-Santamaria (2021) **[34]**	Mixed	1.79	1.1–2.8	0.01	2.11	1.27–3.49	0.003	3.08	1.7–5.5	<0.01	3.63	1.92–6.85	<0.001	Concomitant prescription of opioids	ICI monotherapy or doublet therapy (ipilimumab, nivolumab, pembrolizumab), dose NR	Multicenter retrospective study from three hospitals of adults with locally advanced or metastatic cancer receiving at least three doses of an ICI, *n* = 132 total (Spain), 2015–2018
Gaucher et al. (2021) **[35]**	Mixed	2.60	1.59–4.34	<0.001	1.63 for worse TR with opioid use	0.88–3.06	0.123	1.82	1.40–2.37	<0.001	1.33	0.99–1.79	0.057	Concomitant ICI-opioid use or initiation within 60 days after ICI therapy	ICI monotherapy or doublet therapy (ipilimumab, nivolumab, pembrolizumab), dose NR	Retrospective, single-center of adults receiving ICI age over 18 from various medical departments, *n* = 372 (France), 2010–2019
Botticelli et al. (2021) **[15]**	Metastatic solid tumours (mixed)	1.69	1.37–2.09	<0.0001	1.44	1.15–1.79	0.001	1.6	1.26–2.02	<0.001	1.24	0.97–1.61	0.087	Concomitant opioid use during immunotherapy (strong opioids included only)	nivolumab (240 mg biweekly IV) or pembrolizumab (200 mg every 3 weeks IV) or atezolizumab (1200 mg every 3 weeks) or avelumab (800 mg biweekly)	Multicenter retrospective study from patients who received immunotherapy, *n* = 193 total (Italy), 2014–2019
Kostine et al. (2021) **[17]**	Mixed	1.58	1.23–2.03	<0.001	NR	NR	NR	2.69	2.15–3.38	<0.001	NR	NR	NR	Morphine (1 month before or after the first administration of ICI)	nivolumab, pembrolizumab, atezolizumab, durvalumab, ipilimumab (dose NR) monotherapy or combined ICI regimens (dose NR)	Retrospective cohort of adult patients receiving ICIs, *n* = 635 total (France), 2015- 2017
Fukuokaya et al. (2022) ^#^ **[36]**	Metastatic urothelial carcinoma	NR	NR	NR	NR	NR	NR	NR	NR	NR	2.85	1.08–7.53	0.034	Any opioid prescription within one month before and after the administration of pembrolizumab	pembrolizumab 200 mg IV infusion every 3 weeks	Retrospective cohort study with platinum-treated metastatic UC also treated with second-line pembrolizumab *n* = 227 (Japan), 2018–2021
Gandara et al. (2025) **[24]**	Mixed advanced or metastatic cancers	NR	NR	NR	NR	NR	NR	NR	NR	NR	1.32 worse OS with opioid prescription within ICI monotherapy	1.25–1.40	<0.001	Opioid prescription within 2 months pretherapy	Anti-PD-1/PD-L1 monotherapy	Multicenter retrospective across 280 cancer clinics, *n* = 8440 ICI-monotherapy cohort (USA), 2011–2022
Varghese et al. (2022) ^†^ **[37]**	Mixed	44.7 vs. 16.0 months lower PFS with chronic opioid use	NR	<0.001	NR	NR	NR	42.7 vs. 16.2 months lower OS with chronic opioid use	NR	<0.001	NR	NR	NR	Opioid prescriptions filed from days prior to and 30 days after the end of ICI therapy, binned to chronic vs. occasional opioid use	Anti-PD-1/PD-L1/CTLA-4	Retrospective, single-center cohort *n* = 869 total (USA), 2020–2021
Weinfeld et al. (2022) **[38]**	Unspecified	36 vs. 17 vs. 25 median PFS months with no, low, or high-dose opioids	No opioids (16, -), low-dose opioids (11–38), high-dose opioids (7, -)	NR	NR	NR	NR	37 vs. 18 vs. 10 median OS months with no, low, or high-dose opioids	No opioids (29, 52), low-dose opioids (11,74), high-dose opioids (5, 33)	0.0515	NR	NR	NR	No opioids, low-dose opioids (<60 MME), high-dose opioids (≥60 MME)	Any ICI treatment	Retrospective cohort who received an ICI *n* = 212 total (USA), 2015–2021
Scheff et al. (2023) **[20]**	Recurrent or metastatic HNSCC	NR	NR	NR	1.02, worse PFS per 10 MME/day increase	0.99–1.06	0.200	NR	NR	NR	1.04 worse OS per 10 MME/day increase	1.01–1.08	0.013	MME per day captured prior to and day of ICI therapy	Anti-PD-1 mAb as either front line or second line therapy (dose NR)	Retrospective cohort treated at a cancer center, *n* = 66 total (USA), 2015–2020
Iida et al. (2024) **[39]**	Advanced or metastatic urothelial carcinoma	2.39	1.54–3.72	<0.001	1.30 for worse PFS with opioid use (*p* = 0.266)	0.82–2.07	0.266	3.79	2.32–6.19	<0.001	1.94 for worse OS with opioid use	1.14–3.28	0.014	Any opioid history within 30 days prior and after initiation of ICI therapy	pembrolizumab as a second-line therapy or beyond (200 mg every 3 weeks or 400 mg every 6 weeks)	Multicenter retrospective study at several hospitals *n* = 143 total (Japan), 2018–2021
Kavgaci et al. (2024) ^†^ **[40]**	Mixed recurrent or metastatic cancer	0.59	0.44–0.79	<0.001	0.62 for improved PFS without opioid use	0.49–0.80	<0.001	0.60	0.45–0.80	<0.001	0.58 for improved OS without opioid use	0.43–0.79	<0.001	Patients using morphine, oxycodone, oxymorphone, hydromorphone, fentanyl, codeine, or tramadol for cancer pain management	First-, second- or beyond treatment of nivolumab, atezolizumab, pembrolizumab, avelumab	Retrospective study of patients with advanced cancer who received ICI *n* = 375 total (Turkiye), 2018–2023
Wang Y, Wu Z et al. (2024) **[41]**	Metastatic advanced digestive tract cancer	1.0	0.8–1.4	0.728	NR	NR	NR	1.5	1.1–2.0	0.011	1.5	1.1–2.0	0.010	Any opioid prescription within 30 days prior to initiating and throughout the duration of ICI therapy	toripalimab 240 mg biweekly, sintilimab 200 mg biweekly, camrelizumab 200 mg biweekly, nivolumab 3 mg/kg biweekly, pembrolizumab 200 mg every three weeks, tislelizumab 200 mg every 3 weeks by IV	Multicenter retrospective study of patients with advanced digestive tract cancer treated with ICI alongside antiangiogenic agents *n* = 352 (China), 2019–2022
Prinzi et al. (2025) ^§^ **[26]**	Advanced biliary tract cancer	0.52	0.33–0.82	0.0055	0.91	0.57–1.47	0.72	0.40	0.22–0.73	0.003	0.83 analgesics vs. no analgesics	0.43–1.59	0.58	Analgesics: receiving NSAID and or opioid prior to the start of CGD therapy	ICI combination therapy (CGD): durvalumab (1500 mg IV), gemcitabine (1000 mg/m^2^), and cisplatin (25-day cycle, for up to 8 cycles, followed by durvalumab monotherapy (1500 mg every 4 weeks) until disease progression or unacceptable toxicity	Multicenter retrospective *n* = 493 (patient comedication subgroup) patients with advanced biliary tract cancer (Italy, Germany, Austria, Spain, Belgium, Portugal, UK, USA, Korea, China, Hong Kong, Japan)
Tsuboi et al. (2025) **[29]**	Advanced or metastatic urothelial carcinoma	NR	NR	NR	NR	NR	NR	NR	NR	NR	1.74 worse OS with opioid use	1.46–2.07	<0.001	Any concomitant opioid use at start of ICI therapy	Any ICI treatment	Meta-analysis of total 16 studies (3 prospective and 13 retrospective), concomitant opioid with ICI subgrouped with *n* = 1314 patients

Lung cancer studies have been grouped in the top half of the table, and are separated from the other cancer types by the blue line. Hazard ratios (HR) greater than 1 indicate worse outcomes with opioid exposure unless otherwise stated. Endpoints other than PFS are indicated within table. ^†^ Effect estimate is oriented opposite to the rest of the table: the comparison uses non-opioid (or lower/occasional) use as the index group, or lists the better-survival value first, so an HR <1 (or the first-listed median) corresponds to the non-opioid group. Direction of effect (opioid exposure associated with worse outcome) is unchanged. ^‡^ Adjusted PFS and OS estimates use opposite reference categories (PFS HR expresses opioid use as the exposure, HR greater than 1; OS HR expresses opioid use as the reference, HR <1). ^§^ Exposure is analgesics (NSAID and/or opioid combined), not opioids alone, and the reference category is analgesic. HRs <1 therefore correspond to no-analgesic use. Values are not specific to opioids. ^#^ Progression endpoint in this study was immune progression-free survival (iPFS) rather than conventional PFS. ^¶^ Pooled estimate from a meta-analysis, the univariate/multivariate distinction does not apply. **Abbreviations**: CGD = cisplatin, gemcitabine and durvalumab; CI = confidence interval; DOT = duration of therapy; HNSCC = head and neck squamous cell carcinoma; HR = hazard ratio; IV = intravenous; MEDD = morphine equivalent daily dose; MM = malignant melanoma; MME = morphine milligram equivalents; NR = not reported; NS = non-significant; NSCLC = non-small cell lung cancer; ORR = overall response rate; RCC = renal cell carcinoma; TR = tumour-response; TTF = time-to-treatment failure; TTNT = time-to-next-treatment; UC = urothelial carcinoma; XR = extended release.

### 3.2. In Vivo Preclinical Evidence

Although opioids demonstrate mixed effects on tumour growth and metastasis in rodent models [42], exogenous opioids have consistently been shown to depress anti-cancer immunity through combined direct actions on immunocytes as well as centrally mediated effects involving the hypothalamic–pituitary–adrenal axis and the sympathetic nervous system [43]. Indeed, opioids have been shown to reduce NK cell activity, which is crucial in eliminating cancer cells [44]. Emerging evidence suggests that opioids may also suppress anti-tumour immunity and promote tumour progression through immune checkpoint-associated mechanisms. In murine tumour models, systemic morphine administration accelerates tumour growth and reduces survival, concomitant with impaired CD8^+^ T-cell cytotoxicity, reduced splenic lymphocyte counts, diminished toll-like receptor signaling, and transcriptional upregulation of immune checkpoint-related genes indicative of T-cell exhaustion [45,46]. Importantly, peripheral MOR antagonism using agents such as naldemedine or methylnaltrexone reverses tumour- or morphine-induced immune suppression in vivo, restoring lymphocyte populations, reducing immune checkpoint gene expression, and significantly suppressing tumour progression without abolishing central analgesia [45]. The effect of the endogenous opioid peptide methionine-enkephalin has previously been shown to be distinct from that of exogenous opioids, as it promotes anti-tumour immunity [47,48]. Of note, metenkephalin activates not only opioid δ- and µ-opioid receptors, but also the opioid growth factor receptor (OGFr), which is distinct from the classical GPCR opioid receptors. Complementary animal studies demonstrate that metenkephalin enhances CD8^+^ T-cell expansion and cytotoxic effector molecule expression in tumour-bearing mice, while concurrently increasing PD-1 and CTLA-4 expression, indicating its ability to dynamically regulate both T-cell activation and immune checkpoint engagement in vivo [49]. Collectively, these in vivo data support a model in which exogenous opioids promote immune checkpoint dominance and functional T-cell exhaustion within the tumour microenvironment, whereas MOR inhibition or opioid-sparing strategies preserve anti-tumour immunity and may enhance responsiveness to ICI-directed therapies.

### 3.3. In Vitro and Ex Vivo

Opioids directly influence cell behaviour in experiments carried out in vitro. Morphine increased expression of PD-L1 protein in two human lung cancer lines [46]. In another study, the MOR-inactive but TLR4-active morphine metabolite, morphine-3-glucoronide (M3G), increased PD-L1 protein expression in lung cancer cell lines in a TLR-4 dependent manner [50]. In CD8^+^ T cells isolated from mouse spleen, methionine-enkephalin (MENK), an endogenous opioid peptide able to activate mu and delta ORs in addition to OGFr, was shown to upregulate mu and delta opioid receptors, and increased CD8^+^ T increased proliferation and granzyme production. MENK also promoted PD-1, CTLA-4, CD28 and FasL cell surface expression on T-cells [49]. Together, these in vitro and ex vivo findings establish that opioids exert direct, cell-intrinsic effects on both cancer and immune cells, mediated via combined opioid and non-opioid receptors, providing a mechanistic basis for opioid-driven immune checkpoint dominance observed in vivo.

## 4. Mechanistic Basis of Crosstalk Between Opioids and the Immune Checkpoint Axis

Evidence of direct binding between opioids and canonical immune checkpoint proteins remains sparse. Oxymorphone was shown to bind to the PD-1 homologue V-domain immunoglobulin suppressor of T-cell activation (VISTA, also called PD-1H) using virtual screening and molecular simulation [51]. Oxymorphone demonstrated stable binding within the VISTA pocket across molecular dynamics simulations, with greater conformational stability than the reference inhibitor CA-170. Of a library of 2315 FDA-approved drugs, the top 10 predicted binders further included hydromorphone, morphine, naloxone and diamorphine (heroin). These opioids share a morphinan-based structure and stereochemistry with oxymorphone [51]. While these findings do not establish functional inhibition in biological systems, they suggest a potential functional relationship between opioids and T-cell immunoregulation through VISTA interaction. Furthermore, the homology of VISTA with PD-1 opens the possibility that opioids may interfere with PD-1 function, but this has not been explored yet.

**Figure 2:**
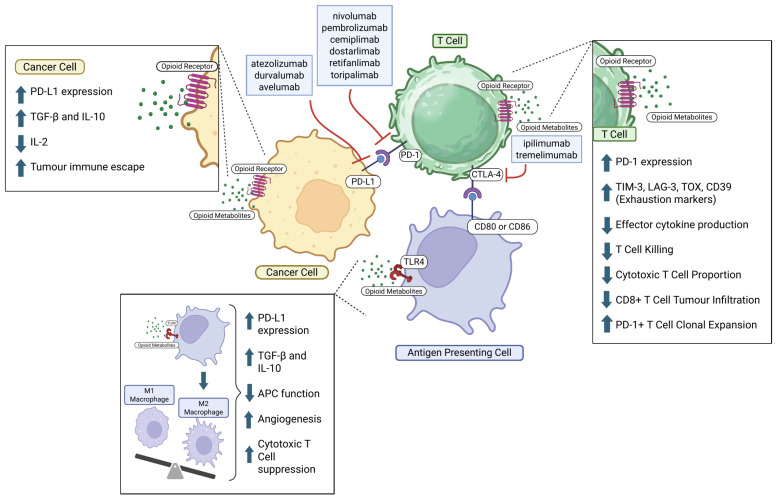
Interaction between opioid signaling and the PD-1/PD-L1 immune checkpoint axis. Opioids and their metabolites act on cancer cells, T cells, and antigen-presenting cells/macrophages via opioid receptors and non-opioid receptors including TLR4 to modulate PD-L1 expression, T-cell exhaustion, and the tumour immune microenvironment. *Created in BioRender. Alan, P. (2026) https://BioRender.com/sitdt8a*.

In addition to direct binding, there is evidence that opioid stimulation modulates the expression of PD-1 or PDL-1 (Figure 2; Table 2).

Additional mechanistic evidence arises from regulation of checkpoint expression. Preclinical models demonstrate that opioids and their metabolites can induce PD-L1 expression on cancer cells. For example, in non-small cell lung cancer models, morphine-3-glucuronide (M3G) upregulated PD-L1 via TLR4-dependent activation of PI3K/Akt and NF-κB signaling, resulting in impaired cytotoxic T-cell-mediated tumour killing [50]. Parallel studies show that morphine itself induces PD-L1, TGF-β, and interleukin (IL)-10 expression through Nrf2/PTEN/MAEL signaling in lung cancer cells, promoting tumour growth and suppressing CD8^+^ T-cell immunity in vivo [46]. Together, these data show that opioid exposure can reinforce adaptive immune resistance through checkpoint upregulation and immunosuppressive cytokine production.

Opioids are known to exert biological effects directly onto immune cells. MOR expression is upregulated in activated CD8^+^ T cells, which increases their responsiveness to opioid ligands [49,54]. Morphine treatment has been shown to promote transcriptional repertoires typical of T-cell exhaustion (such as increased expression of PD-1, TIM-3, LAG-3, TOX, and CD39 and reduced effector cytokine production). In tumour-bearing mice, this results in MOR-dependent decrease in CD4^+^ and CD8^+^ T-cell infiltration and impaired anti-PD1 efficacy [54].

Another mechanism may involve opioids modulation of the innate immune system although the literature is inconsistent on the effect of opioid agonism on macrophage polarization in the context of tumours [58,59,60]. M2 macrophages, which share functional similarities with tumour-associated macrophages, are associated with immunosuppression and angiogenesis. In a clinical study of gastric cancer surgical patients receiving anti-PD-1 treatment, the opioid-free anaesthesia group had higher levels of IL-12, IL-1β, tumour necrosis factor (TNF)-α, and CD68^+^CD163^-^ macrophages but lower levels of IL-10, transforming growth factor (TGF)-β, and CD68^+^CD163^+^ macrophages compared to opioid-based anaesthesia patients, suggesting that opioids promoted M2 and decreased M1 macrophage polarization in the perioperative context [60]. M2-like tumour-associated macrophages are a prominent source of PD-L1 expression in the tumour microenvironment which may exacerbate PD-1/PD-L1 axis-mediated T-cell exhaustion [61,62]. Therefore, opioid-driven macrophage reprogramming represents a plausible additional mechanism by which opioid exposure may attenuate the efficacy of PD-1/PD-L1 blockade, particularly in immunologically active contexts.

In addition to therapeutic opioids administered to patients, endogenous opioids add further complexity to the crosstalk between opioids and checkpoint inhibition. Although largely anti-tumour in animal models, MENK, an endogenous opioid peptide that binds both MOR and DOR, as well as OGFr on tumour cells, also promotes tolerance [63]. In vitro, MENK was shown to activate CD8^+^ T cells, increase activation markers, but also increase PD-1 expression through MOR and DOR agonism [49]. However, in another study, colorectal cancer-bearing mice treated with MENK showed decreased mRNA expression of several immune checkpoints including PD-1 and PD-L1 in tumour tissue, increased tumour infiltration of cytotoxic and helper T cells and promotion of M1-type macrophages with reduced numbers of M2 macrophages. Of note, OGFr was upregulated by MENK in that study [53]. Differences in PD-1 expression between these two studies with MENK treatment could be explained by the duration of exposure to MENK, cell type and receptor engagement profile.

Also responding to opioids, TLR4 emerges as a convergence point linking opioids and their metabolites, immune activation, and checkpoint regulation. Opioids are known to weakly activate TLR4, but to robustly prevent agonist-induced TLR4 activation, suggesting a context-dependent modulation of TLR4 signaling [64,65]. While TLR4 activation in antigen-presenting cells promotes immune priming and PD-L1 expression as part of immune homeostasis, opioid-driven TLR4 signaling appears to skew this response toward immune exhaustion and checkpoint-mediated suppression in cancer-cell contexts [50,57]. Modulation of TLR4 signaling by opioid antagonists further supports its role as a regulatory node connecting opioid signaling tone to checkpoint biology [45,52].

At a systems level, transcriptomic and bioinformatic analyses reinforce a mechanistic antagonism between opioid signaling and immune checkpoint inhibition. In triple-negative breast cancer, opioid-associated gene expression networks overlapped extensively with anti-PD-L1 response signatures but were regulated in opposing directions, particularly within cytotoxic T-cell effector pathways [55]. Similarly, pan-cancer analyses demonstrate that MOR expression is associated with immunologically cold tumour microenvironments characterized by low CD8A and low PD-L1 expression, poorer survival outcomes, and reduced responsiveness to immunotherapy-relevant immune infiltration [56].

Collectively, these studies provide a biological explanation for clinical observations linking opioid exposure with inferior outcomes under PD-1/PD-L1 blockade. Rather than uniformly suppressing immunity, opioids appear to selectively counteract the effector programs restored by checkpoint inhibition through PD-L1 induction on cancer cells, exhaustion of CD8^+^ T cells, and reprogramming of the tumour immune microenvironment. This framework helps explain why traditional cancer cell-centric biomarkers such as PD-L1 expression or tumour mutational burden may lose predictive power in opioid-treated populations, with host-level inflammatory and immune parameters emerging as dominant determinants of immunotherapy response [21,66].

## 5. Opioid Type and Receptor Specificity

The translational value of this field of research would benefit from structure–activity relationship data, since comparisons of the effect of different opioids on checkpoint inhibition (similar to comparison of their overall effect on immunity) is largely missing from in vitro, in vivo or clinical studies.

The ability of PAMORAs to limit the interaction of opioids with ICI efficacy points to the implication of the MOR especially on CD8^+^ T cells. PAMORAs block morphine-induced immunosuppression and recover the anti-tumour efficacy of anti-PD1 in mice. Interestingly, at the dose employed, axelopran (a peripheral mixed inhibitor affecting MOR and KOR more than DOR) was more efficient than methylnaltrexone at reducing the morphine-induced overexpression of PD-L1 on CD8^+^ T cells [54]. In CD8^+^ T cells ex vivo, MENK (an OGFr agonist that also exhibits some OR activity) induced increased PD-1 surface expression on CD8^+^ T cells and this was prevented by CTAP (to inhibit MOR), NT1 (to inhibit DOR) and even more by naltrexone (to inhibit both) [49].

It is noteworthy that MOR antagonists may exhibit an immune checkpoint-mediated protective effect in the absence of opioid agonist administration; the peripheral MOR antagonist naldemedine reversed tumour inoculation-induced overexpression of checkpoint inhibition receptors TIM-3 and LAG-3 (but not PD-1) on CD8^+^ T cells sorted from the spleen [45].

Overall, the structure–activity relationship of opioids in relation to ICI interaction is still to be refined, making recommendations on clinical choice of an opioid on this basis seem premature. What emerges is the potential benefit of adding a peripheral antagonist to a centrally acting opioid analgesic.

## 6. Additional Associations Between Immune Checkpoint Blockade and Opioid Exposure: Pain, Microbiome, and Adverse Events

Beyond their established immunomodulatory roles, immune checkpoint mediators have emerged as direct regulators of pain signaling, with important implications for cancer pain management and opioid responsiveness. PD-1-mediated analgesia intersects mechanistically with opioid signaling at the level of sensory neurons. PD-1 is expressed in neurons of the dorsal root ganglia (DRG), spinal cord, and higher pain-processing centers, where its activation suppresses neuronal excitability and synaptic transmission through SHP-1-dependent signaling pathways, resulting in potent antinociceptive effects [67]. PD-1 is co-localized with the μ-opioid receptor (MOR) in DRG neurons, and PD-1 signaling modulates MOR function and downstream antinociceptive pathways [67,68,69]. In rodent pain models, PD-1 agonism synergistically enhances morphine-induced analgesia, whereas pharmacologic PD-1 blockade using nivolumab attenuates morphine-mediated analgesia and induces hyperalgesia, indicating a direct neuropharmacological interaction that is independent of tumour-directed immune effects [68]. Mechanistically, both nivolumab-mediated PD-1 inhibition and genetic Pd1 deletion have been shown to reduce centrally mediated GABAergic inhibitory signaling through reduced ERK phosphorylation [70]. Consistent with this bidirectional regulation, PD-1 agonism and Pd1 deficiency exert opposing effects on MOR transcription in murine DRG neurons, increasing and decreasing MOR mRNA expression, respectively [69].

Clinical observations, however, remain heterogeneous. A retrospective multicenter study reported reductions in pain intensity shortly after initiation of ICI therapy in cancer patients already receiving opioid analgesics [71]. Pain assessments in this study were conducted within days of ICI initiation, a timeframe unlikely to capture tumour-mediated analgesic effects, and these findings should therefore be interpreted cautiously. In contrast, a multicenter prospective study of NSCLC patients found that those receiving neoadjuvant chemo-immunotherapy required greater postoperative opioid consumption and experienced a higher incidence of “moderate-to-severe pain” three days following surgery compared with chemotherapy alone [72]. Consistent with this, a retrospective cohort of patients undergoing radical esophagectomy reported that those receiving 2–3 cycles of preoperative anti-PD-1 therapy had higher postoperative pain at rest and required greater intraoperative and postoperative opioid doses than patients who did not receive immunotherapy [73]. Additional retrospective analyses reported worse postoperative pain among lung cancer patients with PD-L1-positive tumours receiving oxycodone, whereas no significant differences were observed with sufentanil use [74]. Case reports further describe attenuation of opioid analgesia following nivolumab initiation necessitating opioid dose escalation [75], while other retrospective cohorts report improved analgesia in patients receiving concomitant opioid and ICI therapy [71]. Collectively, these findings suggest that immune checkpoint inhibition may alter opioid efficacy in a context-dependent manner, potentially contributing to opioid dose escalation or altered pain phenotypes in selected patients.

Consistent with this neuro–immune crosstalk, ICIs are frequently associated with inflammatory and sensory adverse effects, particularly involving the skin and endocrine organs. Cutaneous immune-related adverse events (irAEs), including rash, pruritus, and dermatitis, are among the most common toxicities of PD-1/PD-L1 blockade and are often accompanied by significant discomfort or pain [67]. Thyroid dysfunction, another common ICI-associated adverse event, may further contribute to fatigue, myalgias, and altered pain perception. Notably, clinical observations suggest that opioid pathway modulation can influence the severity of certain ICI-associated symptoms, as μ-opioid receptor antagonism has been reported to rapidly alleviate severe checkpoint inhibitor-associated pruritus in individual cases [76,77], supporting functional overlap between opioid-regulated sensory pathways and checkpoint-mediated inflammatory signaling.

Opioids have been shown to cause alterations of the gut microbiome (disrupting microbial composition and gut barrier integrity) over long-term exposure in both preclinical and clinical settings [78,79]. Interestingly, the gut microbiota has been shown to modulate responsiveness to PD-1/PD-L1 therapy [80,81]. Opioid-mediated dysbiosis may thus present an additional mechanism linking opioid therapy to altered responses to PD-1/PD-L1 blockade.

Emerging data further suggest that opioid exposure may also be associated with salivary microbial alterations where in stage IV NSCLC, increased salivary abundance of Actinomyces has been associated with poorer survival outcomes, paralleling adverse prognostic associations observed with opioid exposure [27]. These observations raise the possibility that microbial and opioid-mediated immunomodulatory pathways act synergistically or independently to influence immune checkpoint inhibitor efficacy and patient survival.

Finally, although some retrospective cohorts have reported altered frequencies of irAEs among opioid-exposed patients, including an approximately four-fold reduction in thyroid-related irAEs [82], such findings remain hypothesis-generating and may be confounded by disease severity, symptom burden, or treatment intensity. Numerous case reports describe complex adverse events in the setting of concomitant opioid and ICI exposure, including hypophysitis following tramadol use [83], severe pain crises with systemic inflammation following nivolumab and ipilimumab [84], resolution of pembrolizumab-associated cutaneous eruptions following naloxone administration [76,77], and keratitis in patients with prior opioid exposure receiving pembrolizumab [85]. Conversely, at least one clinical cohort reported reduced cutaneous irAEs among opioid-exposed patients [86], underscoring the heterogeneity of clinical observations.

## 7. Concluding Remarks

Opioid signaling intersects with ICI-mediated anti-tumour immunity across several tumour-intrinsic and host-immune axes. Integrating current preclinical and translational data, we propose a set of mechanistic hypotheses whereby exogenous and endogenous opioids may modulate PD-1/PD-L1 expression, myeloid and T-cell function, and the broader inflammatory milieu, including TLR4, to shape responses to immune checkpoint blockade.

It is important to keep in mind that opioids only represent one of several factors that have been shown to modulate the efficacy of ICIs, including corticosteroids, documented to decrease ICI effectiveness when used prior to their initiation, possibly through prevention of CD8^+^ T cell proliferation [87]; the composition of the gut microbiome [80,88], and accordingly, microbiome-disrupting drugs such as antibiotics or proton pump inhibitors [89], or dietary fiber intake [90]; and the timing of administration, due to dependence of the adaptive immune system on circadian rhythm [91,92]. Therefore, despite the abundance of clinical studies that suggest an association between opioid exposure and poorer outcomes with ICIs, these studies are best viewed as signals that prioritize investigation of specific mechanisms rather than as evidence to support changes in analgesic practice. Our review is intended as a hypothesis-generating framework, outlining testable predictions for future mechanistic studies, biomarker-driven cohorts and interventional trials of opioid-sparing or multimodal analgesic strategies in patients receiving ICIs. Clarifying these mechanisms will therefore be essential before any evidence-based clinical recommendations regarding opioid use and immune checkpoint therapy can be made.

Together, these findings support a unified model in which opioids could enhance PD-1/PD-L1-mediated immune suppression by increasing tumour PD-L1 via innate inflammatory signaling and by sustaining peripheral mu-opioid receptor-linked T-cell dysfunction, thereby diminishing the probability and durability of benefit from ICI blockade therapy.

## Figures and Tables

**Table 2:** Summary of opioid correlation with, or modulation effects on, PD-1/PD-L1 expression across experimental and clinical models.

Study	Opioid Treatment/Signaling Modulation	Opioid Dosage	Cell Type/Animal Model/Data Source	Effect on Expression of PD-1 or PD-L1
In vitro/ex vivo studies				
Jiao et al. (2018) **[49]**	Methionine enkephalin (MENK)	MENK optimal concentration reported as 10^−10^ M (48 h exposure)	Mouse CD8^+^ T cells (splenocyte-derived; in vitro activation)	Increased PD-1, reduced by NTX cotreatment
Wang et al. (2021) **[50]**	Morphine-3-glucuronide (M3G); TLR4 pathway modulation with TAK-242 (TLR4 inhibitor) and PI3K pathway modulation with LY294002 (PI3K inhibitor)	M3G 1–20 μM (24 h exposure); significant at 10 and 20 μM	A549 and H1299 non-small cell lung cancer cells; co-culture with cytotoxic T lymphocytes	Increased PD-L1 protein and membrane expression (TLR4- and PI3K-dependent)
Wang Q et al. (2022) **[46]**	Morphine; MAEL pathway (siRNA knockdown)	Morphine 1, 10, 20 μM	A549 and H460 lung cancer cells; BEAS-2B bronchial epithelial cells as comparator	Increased PD-L1 (dose-dependent); reduced by MAEL knockdown
Min et al. (2025) **[52]**	Low-dose naloxone (LDN); TLR4 pathway (TLR4 overexpression and siRNA knockdown)	LDN 1.0 μM (24 h exposure)	Mouse CD8^+^ T cells in transwell co-culture with mouse forestomach carcinoma (MFC) cells	PD-1 protein from co-cultured CD8^+^ T cells unchanged, PD-1 decreased at the transcript level
In vivo animal models				
Wang et al. (2021) **[50]**	M3G; TLR4 pathway modulation with TAK-242 (TLR4 inhibitor) and PI3K pathway modulation with LY294002 (PI3K inhibitor)	Daily i.p. M3G injections 10 mg/kg for two weeks	C57 BL/6 lung tumour model	Increased PD-L1 protein (dose-dependent) from tumour lysates
Gondoh et al. (2022) **[45]**	Naldemedine	Naldemedine 1 mg/kg oral (repeated administration in tumour setting)	C57BL/6 syngeneic lung carcinoma mouse model	PD-1 transcriptional unchanged in splenic CD8^+^ T cells in response to naldemedine
Wang et al. (2022) **[53]**	MENK	MENK 10, 20, 40 mg/kg i.p. once daily	C57BL/6 male mice inoculated with MC38 colorectal tumour model	PD-L1 unchanged with flow cytometry, decreased Pd-1 and Pd-l1 mRNA in tumour tissues
McIlvried et al. (2024) **[54]**	Morphine exposure ± peripheral mu-opioid receptor antagonists (methylnaltrexone, axelopran) ± anti-PD-1	Morphine 10 mg/kg i.p. twice daily for 4.5 days; methylnaltrexone 10 mg/kg i.p.; axelopran 1 mg/kg i.p.	C57BL/6 MOC1 orthotopic oral squamous cell carcinoma mouse model	Increased tumoural PD-1^+^ CD8^+^ T cells
Min et al. (2025) **[52]**	Low-dose naloxone (LDN)	Naloxone 5 mg/kg/day i.p. for 2 weeks	C57BL/6 MFC cell gastric cancer mouse model	PD-1 transcripts reduced in splenic CD8^+^ T cells
Omics/computational studies				
Scarpa et al. (2023) **[55]**	Transcriptomic network analysis of opioid signatures vs. anti-PD-L1 response signatures	N/A	TNBC patient transcriptomes	Opioid-associated network overlaps anti-PD-L1 response genes, regulated in opposing directions
Sun et al. (2023) **[56]**	Pan-cancer OPRM1 (MOR) expression vs. immune microenvironment typing (TMIT)	N/A	TCGA pan-cancer	MOR-high tumours enriched for low PD-L1
Wu et al. (2023) **[57]**	Pan-cancer co-expression/immune correlation analysis (opioid receptors, OGFr, TLR4)	N/A	TCGA/GEO pan-cancer	TLR4 positively correlated with PD-L1 and CTLA-4 in some cancers; OGFr negatively correlated with PD-L1 in some contexts

## Data Availability

No new data were created or analyzed in this study. Data sharing is not applicable to this article.
